# The impact of inhalation versus total intravenous anesthesia on the immune status in patients undergoing breast cancer surgery: a double-blind randomized clinical trial (TeMP)

**DOI:** 10.3389/fonc.2024.1401910

**Published:** 2024-07-26

**Authors:** Kristina Kadantseva, Valery Subbotin, Roman Akchulpanov, Levan Berikashvili, Mikhail Yadgarov, Lyudmila Zhukova, Guram Kvetenadze, Oxana Svitich, Polina Kukina, Ivan Kuznetsov, Mariya Shemetova, Anastasiya Smirnova, Petr Polyakov, Andrey Shebankov, Valery Likhvantsev

**Affiliations:** ^1^ Department of Clinical Trials and intelligent IT, Federal Research and Clinical Centre of Intensive Care Medicine and Rehabilitology, Moscow, Russia; ^2^ Department of Intensive Care and Anesthesiology, A. Loginov Moscow Clinical Scientific Center, Moscow, Russia; ^3^ Department of Immunology, Mechnikov Research Institute of Vaccines and Sera, Moscow, Russia; ^4^ Federal State Autonomous Educational Institution of Higher Education “N.I. Pirogov Russian National Research Medical University” of the Ministry of Health of the Russian Federation, Moscow, Russia; ^5^ Department of Intensive Care and Anesthesiology, I. Sechenov First Moscow State Medical University, Moscow, Russia

**Keywords:** anesthesia, cancer, immunity, propofol, sevoflurane

## Abstract

**Background:**

Breast cancer (BC) mortality primarily stems from metastases rather than the primary tumor itself. Perioperative stress, encompassing both surgical and anesthetic factors, profoundly impacts the immune system, leading to alterations in neuroendocrine pathways and immune functions, potentially facilitating tumor progression and metastasis. Understanding the immunomodulatory effects of different anesthesia techniques is crucial for optimizing perioperative care in patients with BC. The neutrophil-to-lymphocyte ratio (NLR) serves as one of the key indicators of perioperative immune response.

**Objective:**

To compare the effects of inhalation anesthesia (IA) and total intravenous anesthesia (TIVA) on perioperative immune response in BC surgery patients.

**Methods:**

In this randomized, double-blind clinical trial, BC surgery patients were randomized to receive either TIVA with propofol or IA with sevoflurane. The primary endpoint was NLR assessment. Secondary immune parameters measured included natural killer cells, various T cell subsets, B cells, the immuno-regulatory index [T-helpers (CD3+CD4+)/cytotoxic T-cells (CD3+CD8+)], matrix metallopeptidases (MMP-9), complement components, and immunoglobulins, preoperatively and at 1 and 24 hours postoperatively.

**Results:**

The study included 98 patients (IA: 48, TIVA: 50). The baseline characteristics exhibited remarkable similarity across the groups. No significant difference in absolute NLR values was found between IA and TIVA groups at any time point (1 hour: p = 0.519, 24 hours: p = 0.333). Decreased IgA and IgM levels post-surgery suggested potential negative impacts of IA on humoral immunity compared to TIVA. CRP levels increased more by 24 hours (p = 0.044) in IA compared to TIVA. No significant differences were observed in natural killer cells, T cell subsets, B cells, MMP-9 levels or complement components between groups. Significant differences in the immuno-regulatory index between the TIVA and IA groups at one hour postoperatively (p = 0.033) were not maintained at 24 hours.

**Conclusion:**

While there were no notable differences in NLR among the types of anesthesia, the observed disparities in immunoglobulin content and C-reactive protein levels between groups suggest that we cannot dismiss the potential immunosuppressive effects of inhalational anesthesia in breast cancer surgeries. Further investigation needed to clarify the impact of various anesthesia methods on immune function and their implications for long-term cancer outcomes.

## 1 Introduction

The leading cause of mortality in breast cancer (BC) is metastases, not the primary tumor, leading to 30–40% mortality despite the application of surgery, radiation, and chemotherapy (1). Mastectomy and breast resection are primary treatment methods for BC. It is well-established that perioperative stress, associated with neuroendocrine and immune dysfunctions, plays a crucial role in enhancing the survival of circulating tumor cells and minimal residual disease (2). Various clinical studies have demonstrated that anesthesia also contributes to perioperative stress and may influence cancer recurrence and survival (3, 4). It is hypothesized that surgical interventions in oncological patients may induce suppression of cellular immunity, potentially contributing to adverse long-term outcomes. Multiple studies have shown that using inhaled anesthetics during cancer surgery could lead to worse survival outcomes than using the intravenous anesthetic propofol (5–8).

In a 2023 meta-analysis of 14,036 patients, findings indicated total intravenous anesthesia (TIVA) significantly enhances post-surgical oncological prognosis compared to inhalation anesthesia (IA). This was evidenced by improved overall survival, increased recurrence-free survival, and diminished post-operative pathological manifestations among cancer surgery patients (9). This analysis underscores TIVA’s efficacy in fostering more favorable long-term health outcomes in oncological patient care (9). The observed disparities in clinical outcomes may primarily result from the specific effects exerted by anesthetic agents on immune cell populations, notably natural killer (NK) cells, cytotoxic T lymphocytes (CTLs), and T-helper (Th) cells. Furthermore, the influence of anesthesia on the neutrophil-to-lymphocyte ratio (NLR) elucidates the intricate relationship between anesthesia and immune modulation during surgical interventions (10–12). Oncological processes involve alterations in immune system function at both local and systemic levels, as evidenced by blood parameter changes (13). The neutrophil-to-lymphocyte ratio serves as a marker for assessing inflammation levels, prognosticating cancer and other etiologies, particularly in preoperative preparation (14).

NLR is a simple marker in peripheral blood and is used to assess inflammatory response and physiological stress during the peri-operative period (15). Anesthetic technique may influence NLR, thereby modulating the inflammatory response and surgical outcomes (16). Research spanning a variety of cancer types and stages has demonstrated the prognostic importance of the NLR, revealing that higher NLR levels correlate with diminished survival rates (17).

The study aimed to evaluate the relationship between NLR and anesthetic types in patients undergoing BC surgery.

## 2 Materials and methods

### 2.1 Trial design

We conducted a prospective, randomized, double-blind superiority clinical trial. The protocol was approved by the institutional ethics committee (# 2/2021), registered at https://clinicaltrials.gov (NCT04800393). The study was started on the 29 th of March 2022 The randomization was conducted before the surgery, following the signing of informed consent by patients who presumably met the eligibility criteria. This process employed block randomization with variable block sizes ranging from 20 to 40 patients to ensure statistical balance and allocation concealment across study arms. Researchers, in collaboration with a biostatistician, prepared a series of numbered, opaque, and sealed envelopes, each indicating one of the anesthesia methods used (TIVA or IA). Patients were randomly allocated to TIVA or IA groups before induction of anesthesia. In compliance with the double-blind study protocol, patients and outcome assessors were blinded regarding group assignment. The protocols were implemented to maintain the confidentiality of allocation envelopes, preventing unauthorized disclosure of group assignments, thereby upholding the integrity and validity of the study’s findings. The investigators planned and designed this study in accordance with the recommendations of the Declaration of Helsinki (18). The protocol was drawn up in accordance with the recommendations of Spirit 2013 (19). The manuscript adheres to the Consolidated Standards of Reporting Trials (CONSORT) guidelines (20). The CONSORT checklist is provided in the **Supplementary Table S1**.

### 2.2 Study population

During the study period from March 2022 to September 2023, an assessment of surgeries for BC was conducted. All patients scheduled for this type of surgical intervention were evaluated according to the existing inclusion and exclusion criteria. If a patient met eligibility criteria, a bedside evaluation and written informed consent were obtained. The study enrolled participants who voluntarily provided informed consent, within the age range of 45 to 74 years, diagnosed with primary operable BC at stages IA-IIA (T1–2, N0, M0) through cytological verification, without prior chemotherapy. In the study, two post-randomization exclusion criteria were applied: withdrawal of informed consent (refusal to continue participation in the study) and incomplete surgical intervention (non-resected lesions verified in the early postoperative period, 2 weeks post-surgery). Patients with a history of other oncological diseases in other locations, history of substance abuse, or autoimmune diseases were not included in the study. All eligibility criteria are presented in **Supplementary Table S2**.

### 2.3 Outcomes

The primary endpoint was NLR.

The secondary endpoints were matrix metallopeptidase 9 (MMP-9), immuno-regulatory index [T-helpers (CD3+CD4+)/cytotoxic T-cells (CD3+CD8+)], NK cells (CD3-CD16+) in the blood, C-reactive protein (CRP).

Other endpoints included: T cells of blood (CD3 +), T helpers of blood (CD3 + CD4 +), Cytotoxic T cells of blood (CD3 + CD8 +), B cells of blood (CD19 + CD3-), T cells (CD3 +) + B cells (CD19 + CD3 -) + NK cells (CD3-CD16 +) of blood, IgA, IgM, IgG, complement component C3, complement component C4.

All endpoints were assessed at three time points: before induction of anesthesia, 1 hour after and 24 hours after completion of surgery.

### 2.4 Anesthesia

The enrolled patients were not premedicated. Intraoperative monitoring included electrocardiography (ECG), pulse oximetry, and non-invasive arterial blood pressure measurement. Induction of anesthesia in both groups was performed using propofol at a dosage of 1.5–2.2 mg/kg, fentanyl at 3–5 μg/kg, and a muscle relaxant (either rocuronium bromide or cisatracurium). Muscle relaxation was maintained until neuromuscular blockade reached a Train-Of-Four (TOF) count of 10–0%. Tracheal intubation was carried out with a tube of the appropriate size.

After induction and tracheal intubation, patients from both study groups underwent mechanical ventilation in a pressure control ventilation–volume guaranteed mode using General Electric Avance CS2 or General Electric Medical Systems, USA. Ventilation parameters included an oxygen fraction (FiO2) of 35–40%, tidal volume (TV) of 6–8 ml/kg, positive end-expiratory pressure (PEEP) of 5 cm H2O, an inhalation to exhalation ratio (I:E) of 1:2, and a respiratory rate sufficient to maintain normocapnia (35–45 mm Hg).

Within the TIVA group, propofol was administered for anesthetic maintenance with a dosage of 0.1–0.2 mg/kg/min based on the Schnider model. Conversely, the IA group received sevoflurane for maintaining anesthesia levels. Specifically, sevoflurane was administered at an end-tidal concentration of approximately 1 MAC. The gas mixture used consisted of 35–40% oxygen and air with no nitrous oxide (N2O) administered. The flow rates were adjusted to maintain optimal oxygenation and ventilation parameters throughout the procedure. The mean blood pressure was maintained above 60 mmHg. Fentanyl was dosed individually by the anesthesiologist.

From the start of skin suture application, patients were transitioned to assisted ventilation in the pressure support ventilation-pro mode with the following parameters: flow trigger at 0.2 L/min, support pressure necessary to achieve TV of 6–8 ml/kg with a maximum airway pressure not exceeding 35 cm H2O, PEEP of 5–8 cm H2O, and maintaining normocapnia. Extubation of patients was conducted upon reaching TOF 0.95 and higher.

Transfer from the operating room to the postoperative recovery unit or the ward was based on the severity and extent of the surgical intervention, hemodynamic stability, restoration of spontaneous breathing, and absence of the need for oxygen support. For transfer from the operating room to the ward, the patient, post-extubation, had to score 9 or more points on the modified Aldrete recovery scale.

### 2.5 Blood samples

Venous blood samples were collected before the induction of anesthesia, one hour after surgery, and 24 hours following the completion of the surgery. Each blood collection was performed into three tubes: for immunological research with a separating gel or coagulation activator (to obtain serum); for flow cytometry with K2-EDTA (2-substituted potassium salt of ethylenediaminetetraacetic acid); for general blood test with K2-EDTA – 10 ml.

After each blood collection, the tubes with the biomaterial were immediately transported to the clinical immunology laboratory for further determination of the parameters under study.

Multiparametric analysis helps determine the phenotype of cell populations. Flow cytometry analysis was conducted to evaluate various immune cell populations using specific CD markers with a BD FACSCanto II flow cytometer (BD Biosciences, USA). Detection on this flow cytometer occurred through two types of signals: light scattering (forward and side scatter) and fluorescence emission. Data analysis for identifying T cells (CD3+), T-helper cells (CD3+CD4+), cytotoxic T cells (CD3+CD8+), B cells (CD19+CD3-), and NK cells (CD3-CD16+) involved gating procedures that restrict the analysis to signals from cell populations that meet specific morphological and expression (fluorescence) profiles. Fluorescence plots were then generated within the selected gates. Detailed gating strategies and representative flow cytometry plots from several patients are provided in the **Supplementary Figures S1**, **S2** to enhance transparency and validate our data (21).

The concentration of serum proteins CRP, IgA, IgM, IgG, C3, C4 was measured by nephelometry using a “BN ProSpec” laser nephelometer (22) The concentration of MMP-9 in serum was determined by enzyme immunoassay using the Human MMP-9 Quantikine ELISA Kit strictly in accordance with the instructions for the kit (23).

### 2.6 Data collection

For each study participant, a case report form was completed based on comprehensive examination results. This form documented all data in alignment with the preoperative period, including demographic information (age, gender, height, weight), history of comorbidities, and initial laboratory findings; intraoperative period, detailing anesthesia type, laboratory parameters, and intraoperative monitoring data; and postoperative period, noting complications during hospitalization, laboratory parameters. Morphological data were collected, encompassing cancer staging based on various classifications, including TNM staging, molecular subtype, and HER2 status. Subsequently, these data were entered into an electronic database for further analysis.

### 2.7 Statistical analysis

The sample size for this study was determined based on an evaluation of NLR in patients undergoing BC surgery with TIVA or IA, as reported in two prior studies (15, 24). We calculated that a cohort of 130 patients would provide the study with 80% power to detect an effect size of 0.78. This calculation assumes a standard deviation of 1.5 and a two-sided alpha of 0.05. Additionally, it accounts for an anticipated 10% loss due to loss of follow-up and withdrawal of consent.

Due to difficulties in the supply and procurement of certain reagents, the steering committee stopped recruitment on September 4, 2023, after 98 patients had undergone randomization. This decision was made without knowledge of the interim analysis results. We assessed the sample power (superiority) for NLR with a margin (δ) = 1 (25).

All analyses were conducted using IBM SPSS Statistics for Windows, Version 27.0. Armonk, NY, USA: IBM Corp. Continuous variables with normal and skewed distributions were expressed as mean ± Standard Deviation (SD) and median with Interquartile Range (IQR), respectively. Normality of data was evaluated using the Shapiro-Wilk test and histogram analysis.

Dichotomous variables were analyzed using a two-tailed Chi-square test or Fisher’s exact test, with the Fisher-Freeman-Halton extension applied when necessary. The Mann-Whitney U test compared nonparametric continuous variables between independent groups. For paired samples, analysis involved the Friedman test with Dunn’s *post-hoc* test or the Wilcoxon signed-rank test.

All tests were two-sided, with a significance threshold set at p < 0.05.

A logistic regression model with backward stepwise selection (Wald) was used for sensitivity analysis, aiming to identify predictors of the primary outcome and control for baseline imbalances. Variables with a univariate p-value < 0.10 were included in the model, with anesthesia method being a mandatory inclusion. Collinearity and overfitting risks were assessed using stepwise regression and Spearman correlation tests. In multivariate analyses, variables were reported as odds ratios (ORs) with 95% CIs.

We conducted two types of analyses – intention-to-treat and per-protocol (for the primary endpoint) due to the inadvertent inclusion of 3 patients who did not meet the inclusion criteria (stage 0).

## 3 Results

### 3.1 Patients

**Figure 1:**
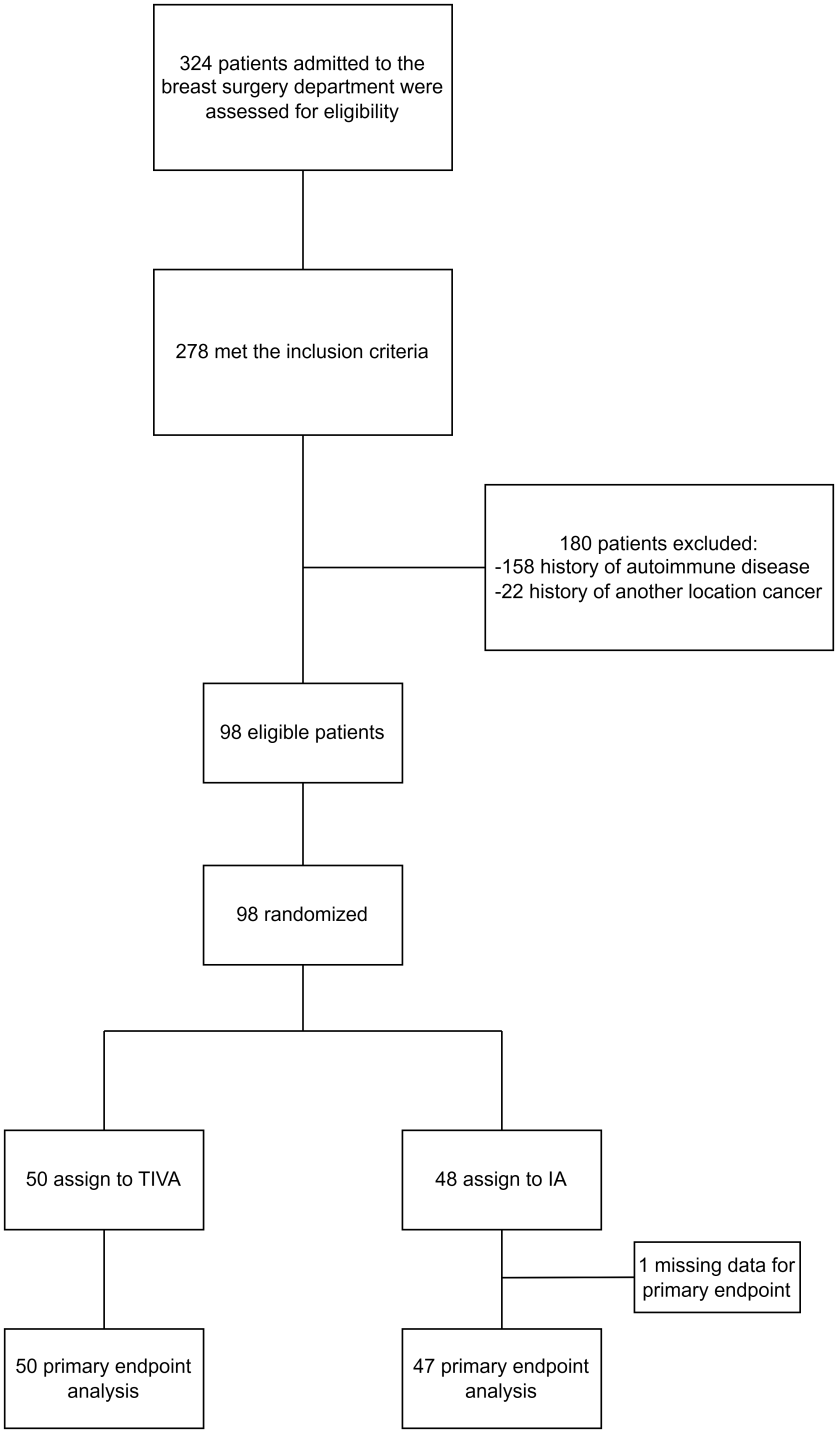
Flow chart of the study.

A total of 324 patients were assessed for eligibility at the A. Loginov Moscow Clinical Scientific Center from March 29, 2022, to September 4, 2023. Of these, 278 met the inclusion criteria; however, 180 were excluded due to autoimmune diseases (158 patients) or a history of cancer at another location (22 patients). Consequently, 98 patients were randomized: 48 to the IA group and 50 to the TIVA group. The primary outcome analysis included 97 patients, with one patient excluded due to missing data from a blood draw error (**Figure 1**).

The baseline patient characteristics, oncological characteristics, type of surgery, and duration of anesthesia were similar between study groups (**Tables 1**, **2**). The median age was 62 years (IQR, 55–68). The majority of patients underwent radical mastectomy (69 patients, 70%). The ICU stay did not exceed 24 hours, and the length of stay in the hospital was 3 days in the IA group (IQR, 3–5) compared to 4 days in the TIVA group (IQR, 3–5), p = 0.131.

**Table 1:** Patients’ baseline characteristics.

Characteristics		IA, N = 48	TIVA, N = 50	p-value
Age, years; N, Me (IQR)		48, 62.5 (56–68)	50, 61 (54–68)	0.545^1^
BMI, kg/m^2^; N, Me (IQR)		46, 29 (23.8–32.0)	50, 27.6 (23.4–31.2)	0.687^1^
Comorbidities, No. (%)				
COVID-19 history		29, 60%	27, 54%	0.521^2^
Uncontrolled diabetes		3, 6.3%	0, 0%	0.114^3^
Chronic obstructive pulmonary disease		1, 2.1%	1, 2.0%	0.999^3^
Cerebrovascular diseases		1, 2.1%	0, 0%	0.490^3^
Peripheral artery diseases		1, 2.1%	0, 0%	0.490^3^
Diabetes		5, 10.4%	2, 4.0%	0.264^3^
Arterial hypertension		30, 63%	28, 56%	0.513^2^
Chronic kidney disease		0, 0%	1, 2.0%	0.999^3^
Coronary artery disease		3, 6.3%	4, 8.0%	0.999^3^
Atrial fibrillation		2, 4.2%	0, 0%	0.237^3^
Arrhythmias		3, 6.3%	1, 2.0%	0.357^3^
Heart failure		6, 12.5%	2, 4.0%	0.155^3^
Liver failure		0, 0%	0, 0%	NA
Dementia		0, 0%	0, 0%	NA
Rheumatologic disease		0, 0%	0, 0%	NA
Peptic ulcer disease		0, 0%	1, 2.0%	0.999^3^
Hemiplegia		0, 0%	0, 0%	NA
Leukemia		0, 0%	0, 0%	NA
Lymphoma		0, 0%	0, 0%	NA
HIV/Hepatitis		0, 0%	0, 0%	NA
Morphometric characteristics				
Tumor site (right)		23, 48%	20, 40%	0.430^2^
TNM classification	T_is_	1, 2.2%	2, 4.0%	0.579^4^
	T1	34, 71%	31, 62%	
	T2	13, 27%	17, 34%	
	N0	48, 100%	50, 100%	NA
	M0	48, 100%	50, 100%	NA
Tumor cell differentiation (G)	G1	8, 17%	10, 20%	0.945^2^
	G2	25, 54%	26, 52%	
	G3	13, 28%	14, 28%	
Stage	0	1, 2.2%	2, 4.0%	0.204^3^
	IА	34, 71%	28, 56%	
	IIА	13, 27%	20, 40%	
Molecular Subtypes	No data	2, 4.2%	1, 2.0%	0.598^4^
	Luminal A	21, 44%	17, 34%	
	Luminal B	23, 48%	28, 56%	
	Triple negative	2, 4.2%	4, 8.0%	
Her2neu+		2, 4.2%	2, 4.0%	0.999^3^

^1^Mann-Whitney test, ^2^chi-square test, ^3^Fisher’s exact test, ^4^Fisher-Freeman-Halton extension. NA, not applicable; IA, inhalation anesthesia; TIVA, total intravenous anesthesia; Me, median value; IQR, interquartile range.

**Table 2:** Surgical and anesthesia characteristics.

Characteristics		IA, N = 48	TIVA, N = 50	p-value
Surgery type	Partial resection	18, 37.5%	11, 22.0%	0.093^2^
	Mastectomy	30, 62.5%	39, 78.0%	
Anesthesia time, min.		87.5 (75.0–102.5)	90.0 (70.0–105.0)	0.839^1^
Surgery time, min.		70.0 (60.0–90.0)	70.0 (60.0–90.0)	0.937^1^
Time of anesthetic administration, min.		85 (75–100)	90 (70–105)	0.908^1^
Intraoperative Fentanyl (mg)		0.3 (0.3–0.4)	0.4 (0.3–0.5)	0.534^1^
Intraoperative propofol (mg)		1.5–2.2 mg/kg-induction of anesthesia No - maintain anesthesia	800 (600–1000)	NA
Intraoperative sevoflurane (MAC)		0.9 (0.8–1.0)	No	NA

^1^Mann-Whitney test, ^2^chi-square test. NA, not applicable; IA, inhalation anesthesia; TIVA, total intravenous anesthesia.

### 3.2 Primary outcome

**Figure 2:**
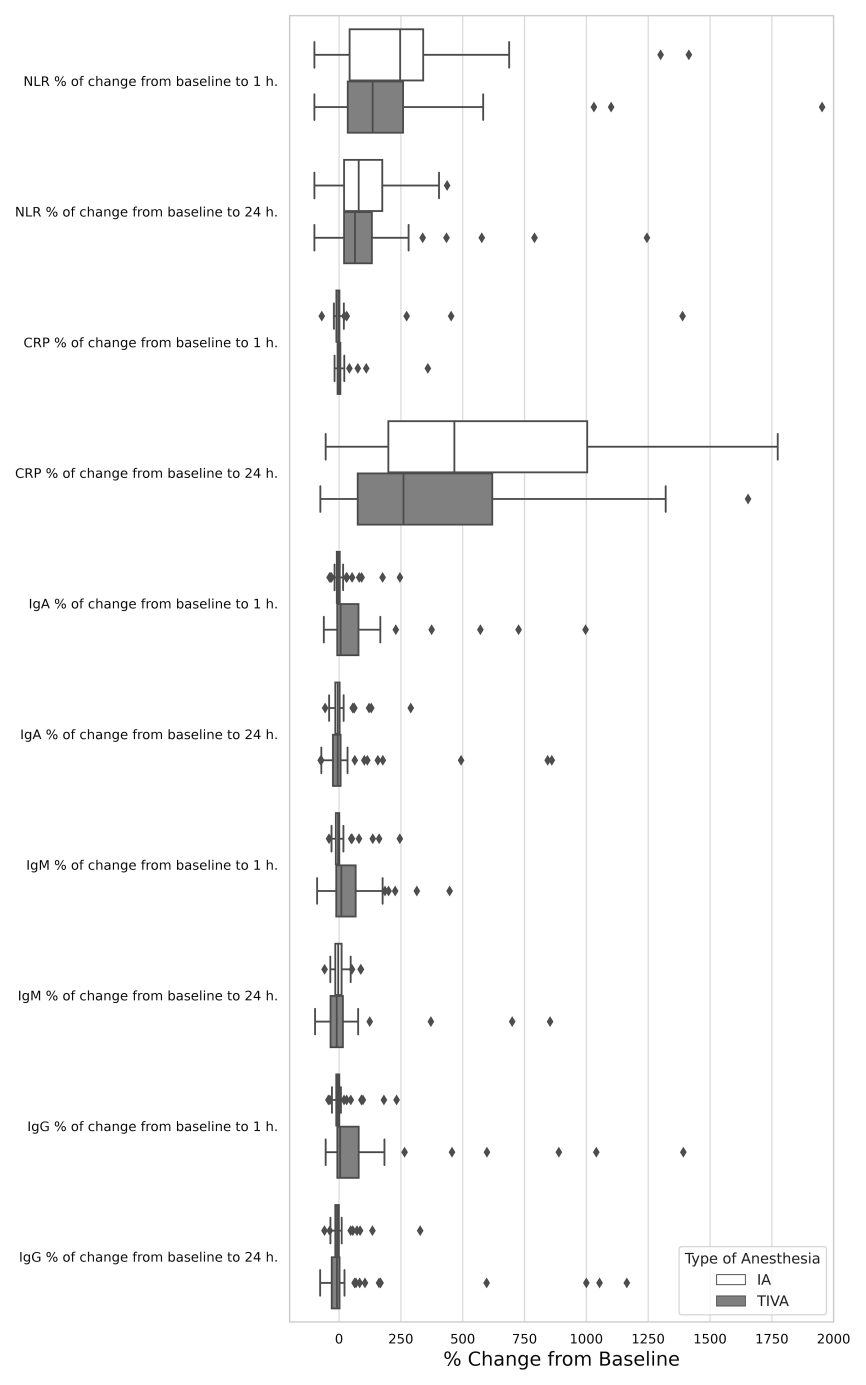
Relative effect of total intravenous anesthesia compared with inhalation anesthesia on serum biomarkers change from baseline to 1 h. and 24 h.

The absolute NLR did not significantly differ between the IA and TIVA groups either pre-operatively (1.7, IQR 1.24–2.43 in the IA group versus 1.65, IQR 1.37–2.17 in the TIVA group, p = 0.767), at 1 hour post-operatively (4.54, IQR 2.40–9.00 versus 4.42, IQR 2.50–6.67, p = 0.519), and at 24 hours post-operatively (3.25, IQR 2.47–4.73 versus 3.00, IQR 2.39–3.64, p = 0.333), as indicated in **Table 3**. No differences between groups were noted in the percentage of change from baseline to 1 hour (p = 0.294) and to 24 hours (p = 0.636, **Figure 2**). Similar results were observed when assessing the relative NLR (**Table 3).**

**Table 3:** Serum biomarkers in patients receiving total intravenous anesthesia vs. patients receiving inhalation anesthesia.

Laboratory parameters		IA, N = 48 N, Me (IQR)	TIVA, N = 50 N, Me (IQR)	p-value^1^
Absolute NLR (abs. neutrophil/abs. lymphocyte)	Preoperative	47, 1.7 (1.24–2.43)	50, 1.65 (1.37–2.17)	0.767
	1 h. postoperative	47, 4.54 (2.4–9)	49, 4.42 (2.5–6.67)	0.519
	% of change from baseline to 1 h.	47, 246.9 (36.4–343.6)	50, 135.7 (34.2–261.1)	0.294
	24 h. postoperative	45, 3.25 (2.47–4.73)	49, 3 (2.39–3.64)	0.333
	% of change from baseline to 24 h.	47, 79.3 (19.6–182.5)	50, 64.4 (18.5–138.8)	0.636
Relative NLR (rel. neutrophil/rel. lymphocyte)	Preoperative	47, 1.69 (1.26–2.39)	50, 1.64 (1.37–2.18)	0.885
	1 h. postoperative	47, 4.5 (2.41–8.5)	49, 5.02 (2.77–6.89)	0.950
	24 h. postoperative	45, 3.25 (2.5–4.64)	49, 3 (2.37–3.65)	0.303
CRP, mg/l	Preoperative	**48, 0.7 (0.3–2.18)**	**50, 1.49 (0.8–3.9)**	**0.023**
	1 h. postoperative	**48, 0.77 (0.32–2.25)**	**50, 1.45 (0.8–3.9)**	**0.038**
	% of change from baseline to 1 h.	48, -3.19 (-11.18–1.4)	50, -0.44 (-6.16–5.26)	0.112
	24 h. postoperative	48, 5.84 (2.49–10.7)	50, 5.54 (2.87–11.4)	0.848
	% of change from baseline to 24 h.	**48, 466 (195–1009)**	**50, 261 (74–631)**	**0.044**
IgA, g/l	Preoperative	**48, 2.33 (1.61–3.04)**	**50, 1.09 (0.47–2.25)**	**<0.001**
	1 h. postoperative	**48, 2.27 (1.56–2.98)**	**50, 1.42 (0.83–2.42)**	**<0.001**
	% of change from baseline to 1 h.	**48, -3.01 (-9.25–2.62)**	**50, 6.22 (-8–81)**	**0.011**
	24 h. postoperative	**48, 2.23 (1.69–2.75)**	**50, 1.11 (0.47–1.95)**	**<0.001**
	% of change from baseline to 24 h.	48, -5.64 (-15.2–2.99)	50, -5.84 (-26.32–6.46)	0.629
IgM, g/l	Preoperative	48, 0.82 (0.51–1.22)	50, 0.74 (0.34–1.06)	0.058
	1 h. postoperative	48, 0.84 (0.61–1.16)	50, 0.72 (0.39–1.2)	0.207
	% of change from baseline to 1 h.	**48, -4.55 (-13.06–0.81)**	**50, 9.3 (-11.91–68.25)**	**0.031**
	24 h. postoperative	**48, 0.81 (0.59–1.16)**	**50, 0.57 (0.36–0.87)**	**0.005**
	% of change from baseline to 24 h.	48, -3.4 (-15.31–10.53)	50, -9.63 (-35.29–17.3)	0.298
IgG, g/l	Preoperative	**48, 10.1 (8.25–12.7)**	**50, 5.76 (2.66–10.6)**	**<0.001**
	1 h. postoperative	**48, 10.05 (8.21–11.7)**	**50, 7.98 (3.97–10.2)**	**<0.001**
	% of change from baseline to 1 h.	**48, -4.77 (-11.73–1.17)**	**50, -4.3 (-7.19–80.5)**	**0.013**
	24 h. postoperative	**48, 9.76 (8.3–11.2)**	**50, 5.64 (2.13–9.91)**	**<0.001**
	% of change from baseline to 24 h.	48, -7.85 (-15.48–0.81)	50, -8.53 (-30.56–2.4)	0.589
C3, g/l	Preoperative	48, 1.25 (1.08–1.36)	50, 1.3 (1.11–1.49)	0.161
	1 h. postoperative	48, 1.2 (1.03–1.35)	50, 1.34 (1.13–1.47)	0.078
	24 h. postoperative	48, 1.23 (1.03–1.39)	50, 1.31 (1.17–1.48)	0.086
C4, g/l	Preoperative	48, 0.31 (0.25–0.35)	50, 0.31 (0.24–0.35)	0.752
	1 h. postoperative	48, 0.3 (0.23–0.33)	50, 0.31 (0.23–0.35)	0.709
	24 h. postoperative	48, 0.3 (0.26–0.34)	50, 0.31 (0.24–0.39)	0.621
MMP-9, ng/ml	Preoperative	29, 19.7 (13.7–20.0)	11, 17.4 (13.4–19.3)	0.254
	24 h. postoperative	29, 20.0 (15.9–20.0)	11, 17.4 (14.7–20.0)	0.492
Absolute neutrophil count, 10^9^/L	Preoperative	47, 3.7 (3.1–4.6)	50, 3.3 (2.5–4)	0.086 0.866 0.217
	1 h. postoperative	47, 5.6 (4–7)	49, 5.4 (4.2–7.2)	
	24 h. postoperative	45, 6.8 (5.3–9.1)	49, 5.8 (4.6–8.6)	
Absolute lymphocyte count, 10^9^/L	Preoperative	47, 2.06 (1.7–2.6)	50, 2.1 (1.5–2.35)	0.237
	1 h. postoperative	47, 1.2 (0.7–1.7)	50, 1.3 (1–1.6)	0.558
	24 h. postoperative	45, 2 (1.8–2.4)	49, 2 (1.6–2.5)	0.604
Relative neutrophil count, %	Preoperative	47, 57.4 (48,4–63.9)	50, 55.45 (51.9–62)	0.865
	1 h. postoperative	47, 77.9 (62.7–87.4)	49, 78.5 (67.2–83.3)	0.789
	24 h. postoperative	45, 69.5 (64.4–74.6)	49, 67.6 (63.5–71.8)	0.440
Relative lymphocyte count, %	Preoperative	47, 33.3 (26.5–39)	50, 33.75 (28.4–38.1)	0.894
	1 h. postoperative	47, 17.2 (10.2–26)	50, 16.4 (11.9–25.5)	0.928
	24 h. postoperative	45. 21.5 (15.8–25.6)	49, 23 (19.6–26.8)	0.269
Т cells (СD3+), %	Preoperative	47, 70.4 (64.9–78.9)	50, 70.75 (62.2–78)	0.829
	1 h. postoperative	47, 67.5 (54.1–74.9)	50, 65.95 (54.9–72.3)	0.549
	24 h. postoperative	47, 73.8 (65.5–81.2)	50, 71.9 (67.2–77.3)	0.654
T helper cell (CD3+CD4+), %	Preoperative	47, 61.5 (53.9–66.8)	50, 59.75 (53.1–70.8)	0.798
	1 h. postoperative	47, 61.1 (53.7–68.2)	50, 56.8 (43.7–69.3)	0.292
	24 h. postoperative	47, 65.7 (56.7–71.5)	50, 63.4 (52.6–72.2)	0.518
Cytotoxic T cell (CD3+CD8+), %	Preoperative	47, 32 (25.3–38.6)	50, 32.65 (22.4–39.4)	0.940
	1 h. postoperative	47, 31 (26–37.6)	50, 36 (23–45.1)	0.392
	24 h. postoperative	47, 27 (22.7–36.2)	50, 30.15 (21.7–38.4)	0.433
Immunoregulatory index (CD4+/CD8+ ratio)	Preoperative	46, 1.85 (1.51–2.46)	50, 1.66 (1.34–3.16)	0.872
	1 h. postoperative	47, 1.95 (1.47–2.52)	50, 1.54 (0.97–2.84)	0.203
	% of change from baseline to 1 h.	**46, 1.58 (-16.00–18.40)**	**50, -8.15 (-35.00–10.16)**	**0.033**
	24 h. postoperative	45, 2.57 (1.68–3.02)	49, 2.13 (1.37–3.01)	0.394
	% of change from baseline to 24 h.	46, 9.08 (-5.78–33.18)	50, 13.39 (-10.35–34.59)	0.924
В cells, %	Preoperative	47, 8.9 (7–11.5)	50, 9.65 (6.2–13.1)	0.762
	1 h. postoperative	47, 10.7 (8.1–14.3)	50, 10.8 (7.7–16.8)	0.991
	24 h. postoperative	47, 12.7 (8.5–15.7)	49, 12.3 (9.1–16.8)	0.484
NK cells, %	Preoperative	47, 16.2 (11.8–23.4)	50, 14.9 (8.7–22.3)	0.191
	1 h. postoperative	47, 16 (10.3–27.9)	50, 17.5 (10.7–25)	0.988
	24 h. postoperative	47, 11.3 (8–16.6)	48, 8.95 (6.45–14.15)	0.189
Т cells (СD3+), В cells (СD19+CD3), NK cells (CD3-CD16+), %	Preoperative	**46, 98.5 (96.6–99.8)**	**50, 97.2 (95.4–98.6)**	**0.021**
	1 h. postoperative	47, 98.3 (95.5–99.9)	50, 97.25 (93.8–99.2)	0.151
	24 h. postoperative	45, 98.4 (96.8–99.4)	48, 97.4 (94.6–98.95)	0.105

^1^Mann-Whitney test. IA, inhalation anesthesia; TIVA, total intravenous anesthesia; Me, median value; IQR, interquartile range. Statistically significant differences are marked with bold font.

### 3.3 Secondary outcomes

Patients in the IA group exhibited significantly lower CRP levels preoperatively (p = 0.023) and 1 hour post-operatively (p = 0.038). However, the increase in CRP from baseline to 24 hours post-operatively was more marked in the IA group (p = 0.044), as detailed in **Table 3**. Levels of MMP-9 and NK cells were comparable in both groups at all assessment time points (**Table 3**). The immunoregulatory index (CD4+/CD8+ ratio) was comparable in both groups at all assessment time points, however, the change from baseline to 1 hour post-operatively was statistically different (p = 0.033), as detailed in **Table 3**.

### 3.4 Other outcomes

IgA and IgG levels were significantly higher in the IA group both before surgery (p < 0.001) and at 1 hour and 24 hours post-operatively (p < 0.001), compared to TIVA group. The IgM level was significantly higher in the IA group at 24 hours after surgery (p = 0.005). Notably, in the IA group, all immunoglobulin levels generally decreased 1 hour post-operatively compared to baseline, whereas in the TIVA group, remained unchanged or increased (IgA: p = 0.11, IgM: p = 0.031, IgG: p = 0.013), **Table 3**, **Figure 2**. Significant differences were also observed in the level of T cells (CD3+)+B cells (CD19+CD3-)+NK cells (CD3-CD16+) pre-operatively (p = 0.021). Levels of C3, C4, absolute and relative neutrophils and lymphocytes, as well as T (CD3+), B, T helper cells (CD3+ CD4+), CTLs (CD3+CD8+) were comparable in both groups at all assessment time points (**Table 3**).

### 3.5 Trends in serum biomarkers levels

Absolute and relative NLR, absolute and relative neutrophil count, and B cells significantly increased at 1 hour and 24 hours from baseline in both groups (p ranging from <0.001 to 0.01, **Table 4**). IgM and IgG levels decreased at 24 hours post-operatively in the IA group. Levels of C3 and C4 also decreased in the IA group at 1 hour post-operatively. T cell (CD3+) levels decreased in both groups at 1 hour post-operatively, while the immunoregulatory index increased at 24 hours in the IA group. All trends in serum biomarker levels are presented in **Table 4**.

**Table 4:** Trends in serum biomarker levels in patients receiving total intravenous anesthesia vs. patients receiving inhalation anesthesia.

Laboratory parameters	IA^1^	TIVA^1^	IA		TIVA	
			Trend from baseline to 1 h.^2^	Trend from baseline to 24 h.^2^	Trend from baseline to 1 h.^2^	Trend from baseline to 24 h.^2^
Absolute NLR	<0.001	<0.001	↑<0.001	↑<0.001	↑<0.001	↑<0.001
Relative NLR	<0.001	<0.001	↑<0.001	↑<0.001	↑<0.001	↑<0.001
CRP, mg/l	<0.001	<0.001	0.248	↑<0.001	0.999	↑<0.001
IgA, g/l	0.006	0.002	0.109	↑0.006	0.401	0.154
IgM, g/l	0.015	0.094	0.065	↓0.028	NA	NA
IgG, g/l	<0.001	0.001	↓0.049	↓<0.001	0.999	↓0.010
C3, g/l	0.001	0.083	↓0.001	0.074	NA	NA
C4, g/l	0.014	0.103	↓0.013	0.459	NA	NA
MMP-9, ng/ml	NA	NA	NA	0.163^3^	NA	0.374^3^
Absolute neutrophil count, 10^9^/L	<0.001	<0.001	↑<0.001	↑<0.001	↑<0.001	↑<0.001
Absolute lymphocyte count, 10^9^/L	<0.001	<0.001	↓<0.001	↓<0.001	↓<0.001	↓<0.001
Relative neutrophil count, %	<0.001	<0.001	↑<0.001	↑<0.001	↑<0.001	↑<0.001
Relative lymphocyte count, %	<0.001	<0.001	↓<0.001	↓<0.001	↓<0.001	↓<0.001
Т cells (СD3+), %	0.001	<0.001	↓0.07	0.540	↓0.028	0.485
T helper cell (CD3+CD4+), %	0.020	0.005	0.999	0.07	0.121	0.750
Cytotoxic T cell (CD3+CD8+), %	0.016	0.004	0.999	↓0.016	0.297	0.297
Immunoregulatory index (CD4+/CD8+ ratio)	0.034	0.003	0.999	↑0.037	0.258	0.258
В cells, %	<0.001	<0.001	↑<0.001	↑<0.001	↑0.010	↑<0.001
NK cells, %	0.001	<0.001	0.999	↓0.016	0.662	↓0.018
Т cells (СD3+), В cells (СD19+CD3), NK cells (CD3-CD16+), %	0.805	0.905	NA	NA	NA	NA

^1^Friedman test, ^2^Dunn’s post-hoc test; ^3^Wilcoxon signed-rank test; NA, not applicable; IA, inhalation anesthesia; TIVA, total intravenous anesthesia. The symbols ↑ and ↓ represent an increase and a decrease in the parameter over time, respectively.

### 3.6 Sensitivity analysis for the primary outcome

For the regression analysis, we categorized patients based on their absolute NLR level at 24 hours (≥3 [median value] and others) and included the following predictors in the analysis: type of anesthesia, baseline CRP, baseline IgA, IgM, IgG, and T cells (CD3+)+B cells (CD19+CD3-)+NK cells (CD3-CD16+), and type of surgery. The results of the univariate and multivariate analyses for the association of baseline variables with the primary outcome confirmed the lack of significant effect for the type of anesthesia (univariate OR: 1.2, 95% CI 0.5–2.7, p = 0.660; adjusted OR: 1.6, 95% CI 0.6–4.2, p = 0.382).

According to the per-protocol analysis, the intention-to-treat result was confirmed, and no significant differences were found between IA and TIVA at all time points (p = 0.982 at baseline, p = 0.715 at 1 hour, and p = 0.429 at 24 hours postoperatively).

### 3.7 Power assessment

The sample power for the primary endpoint (superiority for NLR at 24 hours) for values of 3.25 in the IA group vs. 3 in the TIVA group with a standard deviation of 2, a 5% alpha level, and 97 patients was 83.7%.

## 4 Discussion

### 4.1 Key findings

The study findings reveal a consistent suppression of cellular immunity in both groups, evidenced by decreased lymphocyte levels and increased neutrophil counts postoperatively, both relative and absolute. This led to a uniform elevation of the NLR across all examined time points. The result indicates that the observed changes are attributable to the presence of perioperative stress in patients undergoing BC surgery, rather than to the specific type of anesthetic agent used.

The most significant finding of this study was the suppression of humoral immunity in the IA group, reflected by decreased IgA and IgM levels, whereas these markers were elevated in the TIVA group. These observations highlight the potential immunosuppressive consequences of IA, which could have implications for postoperative recovery and long-term outcomes in oncological settings (26, 27). The postoperative decrease in IgA and IgM levels may impair the body’s specific antibody production, undermining resistance to infectious diseases and potentially impacting long-term oncological outcomes, while simultaneously compromising the production of tumor-specific antibodies essential for opsonization and enhancement of phagocytosis by macrophages and dendritic cells, critical for the effective functioning of the complement system and support of CTLs’ anti-tumor activity (28).

Significant changes in CRP levels from baseline to 24 hours postoperatively in both groups underscore a pronounced inflammatory response, reflecting perioperative stress. However, a more substantial increase was observed in the IA group compared to the TIVA group. This difference may be attributed to the anti-inflammatory properties of propofol and a shift in immune balance towards a cytotoxic anti-tumor response (29, 30). Elevated CRP levels contribute to cancer progression by serving as a marker of disease advancement and enhancing inflammation, which supports tumor growth by aiding DNA damage, angiogenesis, and metastasis (31, 32). Additionally, CRP’s role in pathogen opsonization and complement system activation underscores its involvement in creating a tumor-friendly microenvironment (31–33).

Regarding MMP-9, one of the primary markers indicating adverse progression of BC (34), and cellular immunity parameters, no significant intergroup differences were identified. Moreover, the intragroup dynamics, demonstrating a decrease in T cells (CD3+), NK cells, and an increase in B cells, were comparable across both groups, further confirming the impact of perioperative stress on cellular immunity indicators, regardless of the anesthetic type.

### 4.2 Comparison with previous studies

According to previous studies, exceeding a NLR threshold of 3.3 indicated a decrease in survival rates among patients with BC. (35, 36). Although this parameter exceeded the threshold value in both groups at one hour postoperatively, it returned to lower levels within twenty-four hours. Despite its potential, the study found no significant variance in NLR values between groups, which did not substantiate the hypothesis that IA affects the immune response in BC surgery more than intravenous anesthesia.

The results of this study, demonstrating a more pronounced increase in CRP levels 24 hours postoperatively in the IA group compared to the TIVA group, align with findings from the study in non-cardiac surgery with oncological patients (37). However, the study involving gastric cancer patients has shown no significant differences in CRP levels between the volatile anesthesia and TIVA groups at any postoperative time point (38). This necessitates further investigation to understand the varying impacts of anesthetic techniques on inflammatory responses in patients with cancer.

To the best of our knowledge, in this study, for the first time, suppression of humoral immunity was demonstrated through the impact of IA on the levels of IgA and IgM in patients with BC.

Our results diverge from prior findings that indicated an impact of specific anesthetic techniques, such as sevoflurane-remifentanil and propofol-remifentanil combinations, on cellular immunity in BC surgeries. Contrary to the suppression of NK cell cytotoxicity and other cellular immune responses observed with these specific anesthetic regimens in the referenced study, our data revealed no significant alterations in cellular immune function (24). In a 2020 study, Efremov et al. assessed the effects of IA compared to TIVA on cell-mediated immunity during kidney cancer surgeries, producing findings consistent with our research. It was demonstrated that there were no significant differences in NK cell counts, total T lymphocytes, or T lymphocyte subpopulations between the IA and TIVA groups (39). Our research aligns with previous findings, demonstrating that both TIVA and IA exhibit negligible effects on cellular immune responses. This is evidenced by comparable perioperative levels of circulating natural killer cells, helper T cells (Th1, Th17), and cytotoxic T lymphocytes, thus underscoring the limited impact of anesthetic choice on cellular immunity in the context of oncological surgeries (12, 40).

Contrasting with our study, Likhvantsev et al. (2022) documented post-surgical decreases in MMP-9 for TIVA recipients versus IA (p = 0.030) (41).

### 4.3 Limitations

Despite the study being conducted as a prospective, double-blind trial with unequivocally interpretable laboratory data, it nevertheless had several limitations. First, our study was conducted in line with routine clinical practice, thereby incorporating propofol for induction in the IA group. However, while this approach may have influenced the actual magnitude of the IA effect, it simultaneously enhanced the clinical relevance of the findings. Secondly, cell count measurements might not fully reflect function or actual differences in cytotoxic activity. A significant limitation of the study was the baseline imbalance between groups in levels of CRP, IgA, and IgG, however, the sample power for the primary endpoint exceeded 80%. An additional limitation of our study was the inability to perform analyses for interleukin 6 and phagocytosis, due to supply restrictions for the necessary reagents. Furthermore, there was an imbalance in the number of patients between the IA and TIVA groups assessed for MMP-9, attributed to the relatively large size of the varying block randomization.

### 4.4 Future directions

Further research is necessary to understand IA’s impact on immune function, including cytokine profiles, specific antibodies, and lymphocyte subpopulations. However, our findings prompt reconsideration of IA’s use in oncologic patient surgery, with a preference for TIVA due to propofol’s positive effect on anti-tumor immunity.

## 5 Conclusion

In conclusion, although there were no notable differences in NLR among the types of anesthesia, our study demonstrated that inhalational anesthesia significantly affected humoral immunity post-surgery, while cellular immunity remained largely unaltered. This selective suppression could potentially compromise the patient’s ability to eradicate residual cancer cells and increase the risk of postoperative complications and tumor recurrence. These findings suggest a more cautious approach to the use of IA, particularly in breast cancer surgeries, where maintaining immune function is crucial for long-term disease management and patient survival.

## Data availability statement

The raw data supporting the conclusions of this article will be made available by the authors, without undue reservation.

## Ethics statement

The studies involving humans were approved by A.S. Loginov Independent ethics committee (# 2/2021). The studies were conducted in accordance with the local legislation and institutional requirements. The participants provided their written informed consent to participate in this study.

## Author contributions

KK: Data curation, Methodology, Writing – original draft, Writing – review & editing. VS: Writing – original draft, Writing – review & editing, Supervision, Validation. RA: Data curation, Writing – original draft, Writing – review & editing. LB: Data curation, Formal analysis, Writing – original draft, Writing – review & editing. MY: Data curation, Formal analysis, Writing – original draft, Writing – review & editing. LZ: Supervision, Writing – original draft, Writing – review & editing. GK: Supervision, Writing – original draft, Writing – review & editing. OS: Writing – original draft, Writing – review & editing. PK: Writing – original draft, Writing – review & editing. IK: Writing – original draft, Writing – review & editing. MS: Writing – original draft, Writing – review & editing. ASm: Data curation, Writing – original draft, Writing – review & editing. PP: Writing – original draft, Writing – review & editing. ASh: Data curation, Writing – original draft, Writing – review & editing. VL: Conceptualization, Methodology, Project administration, Writing – original draft, Writing – review & editing.
