# Supplementary material for: The impact of inhalation versus total intravenous anesthesia on the immune status in patients undergoing breast cancer surgery: a double-blind randomized clinical trial (TeMP)
Source: Front Oncol. 2024 Jul 26;14:1401910. doi: 10.3389/fonc.2024.1401910 (PMC11310116; doi:10.3389/fonc.2024.1401910)

Supplementary Material

1 Table S1. CONSORT checklist.

Section/Topic	Item No	Checklist item	Reported on page No
**Title and abstract**			
	1a	Identification as a randomised trial in the title	1
	1b	Structured summary of trial design, methods, results, and conclusions (for specific guidance see CONSORT for abstracts)	2
**Introduction**			
Background and objectives	2a	Scientific background and explanation of rationale	3
	2b	Specific objectives or hypotheses	3
**Methods**			
Trial design	3a	Description of trial design (such as parallel, factorial) including allocation ratio	3
	3b	Important changes to methods after trial commencement (such as eligibility criteria), with reasons	-
Participants	4a	Eligibility criteria for participants	4
	4b	Settings and locations where the data were collected	5-6
Interventions	5	The interventions for each group with sufficient details to allow replication, including how and when they were actually administered	4-6
Outcomes	6a	Completely defined pre-specified primary and secondary outcome measures, including how and when they were assessed	4
	6b	Any changes to trial outcomes after the trial commenced, with reasons	-
Sample size	7a	How sample size was determined	6
	7b	When applicable, explanation of any interim analyses and stopping guidelines	-
Randomisation:			
Sequence generation	8a	Method used to generate the random allocation sequence	3-4
	8b	Type of randomisation; details of any restriction (such as blocking and block size)	3-4
Allocation concealment mechanism	9	Mechanism used to implement the random allocation sequence (such as sequentially numbered containers), describing any steps taken to conceal the sequence until interventions were assigned	3-4
Implementation	10	Who generated the random allocation sequence, who enrolled participants, and who assigned participants to interventions	3-4
Blinding	11a	If done, who was blinded after assignment to interventions (for example, participants, care providers, those assessing outcomes) and how-	3-4

	11b	If relevant, description of the similarity of interventions	-
Statistical methods	12a	Statistical methods used to compare groups for primary and secondary outcomes	6
	12b	Methods for additional analyses, such as subgroup analyses and adjusted analyses	6
**Results**			
Participant flow (a diagram is strongly recommended)	13a	For each group, the numbers of participants who were randomly assigned, received intended treatment, and were analysed for the primary outcome	p.7/Figure 1
	13b	For each group, losses and exclusions after randomisation, together with reasons	-
Recruitment	14a	Dates defining the periods of recruitment and follow-up	-
	14b	Why the trial ended or was stopped	-
Baseline data	15	A table showing baseline demographic and clinical characteristics for each group	p.8/ Table 1
Numbers analysed	16	For each group, number of participants (denominator) included in each analysis and whether the analysis was by original assigned groups	p.8/Table 1
Outcomes and estimation	17a	For each primary and secondary outcome, results for each group, and the estimated effect size and its precision (such as 95% confidence interval)	pp. 11-12/ Table 3
	17b	For binary outcomes, presentation of both absolute and relative effect sizes is recommended	pp. 11-12/ Table 3
Ancillary analyses	18	Results of any other analyses performed, including subgroup analyses and adjusted analyses, distinguishing pre-specified from exploratory	6-12
Harms	19	All important harms or unintended effects in each group (for specific guidance see CONSORT for harms)	-
**Discussion**			
Limitations	20	Trial limitations, addressing sources of potential bias, imprecision, and, if relevant, multiplicity of analyses	15
Generalisability	21	Generalisability (external validity, applicability) of the trial findings	14-15
Interpretation	22	Interpretation consistent with results, balancing benefits and harms, and considering other relevant evidence	14-15
**Other information**			
Registration	23	Registration number and name of trial registry	3
Protocol	24	Where the full trial protocol can be accessed, if available	-
Funding	25	Sources of funding and other support (such as supply of drugs), role of funders	16

Citation: Schulz KF, Altman DG, Moher D, for the CONSORT Group. CONSORT 2010 Statement: updated guidelines for reporting parallel group randomised trials. BMC Medicine. 2010;8:18.
© 2010 Schulz et al. This is an Open Access article distributed under the terms of the Creative Commons Attribution License (http://creativecommons.org/licenses/by/2.0), which permits unrestricted use, distribution, and reproduction in any medium, provided the original work is properly cited.

*We strongly recommend reading this statement in conjunction with the CONSORT 2010 Explanation and Elaboration for important clarifications on all the items. If relevant, we also recommend reading CONSORT extensions for cluster randomised trials, non-inferiority and equivalence trials, non-pharmacological treatments, herbal interventions, and pragmatic trials. Additional extensions are forthcoming: for those and for up-to-date references relevant to this checklist, see www.consort-statement.org.

2 Table S2. Inclusion and exclusion criteria.

Inclusion criteria:	Exclusion criteria:
1. The age of the patients is from 45 to 74 years;<br>2. Primary operable breast cancer (BC) without prior chemotherapy;<br>3. Cytologically confirmed breast malignant tumor of IA-IIA stages (T1-2, N0, M0);<br>4. Signed informed consent.	1. Acute cerebrovascular accident (CVA) occurred in the previous 6 months;<br>2. Myocardial infarction (MI) occurred in the previous 6 months;<br>3. Acute arterial thrombosis occurred in the previous 6 months;<br>4. Acute venous thromboembolism occurred in the previous 6 months;<br>5. Subarachnoid hemorrhage occurred during the previous 3 months;<br>6. Chronic kidney disease (CKD) stage 3B-5;<br>7. Chronic heart failure (NYHA) class 3-4;<br>8. Pregnancy;<br>9. History of another location cancer;<br>10. History of drug addiction;<br>11. Autoimmune diseases in history;<br>12. Post-randomization: withdrawal of informed consent (refusal to continue participating in the study);<br>13. Post-randomization: surgical intervention, performed not in full (verified non-resected lesions in the early p/o period, 2 weeks p/o).

3. Figure S1. Flow Cytometry gating. Patient 1.

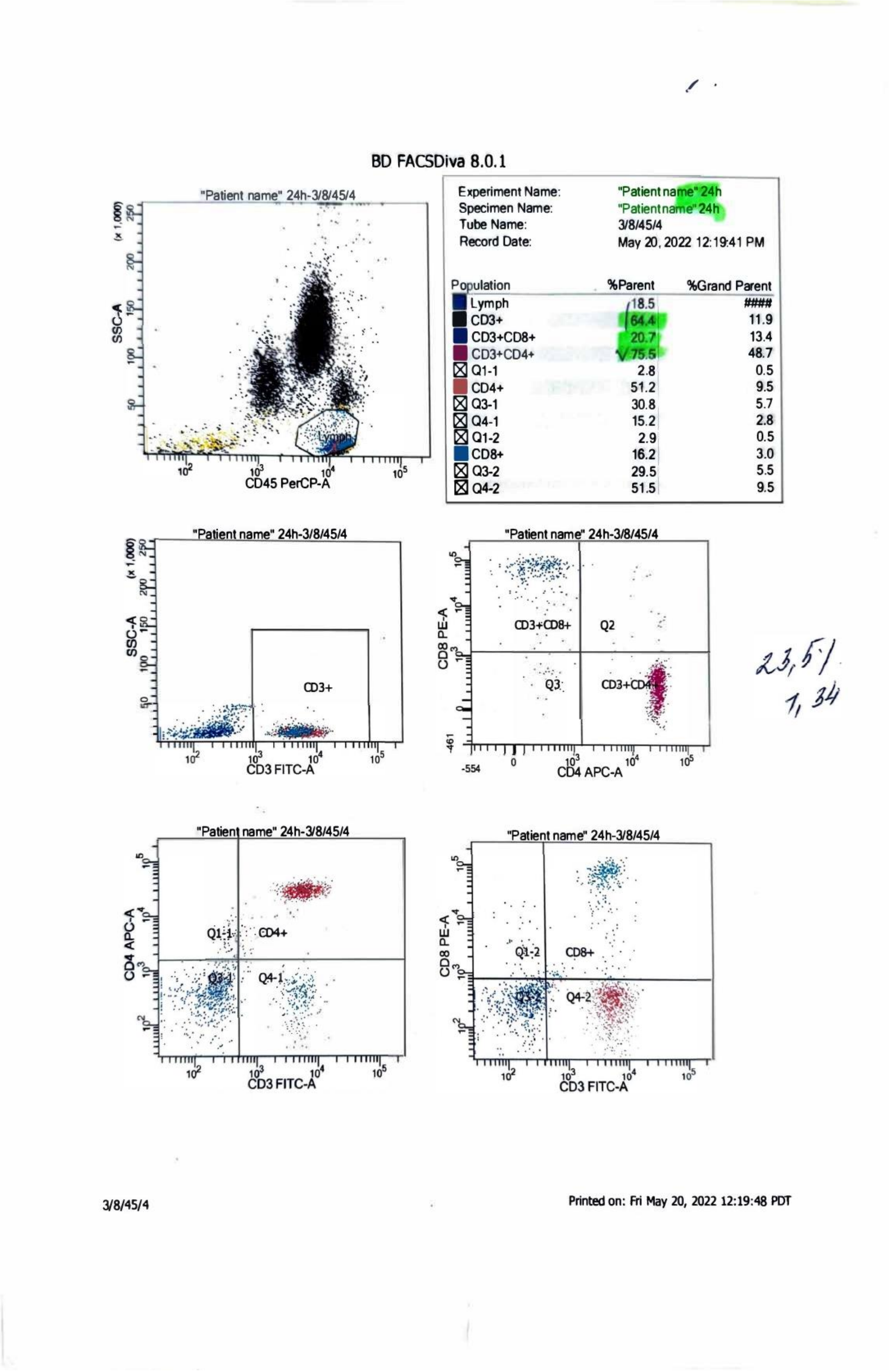

Population	%Parent	%Grand Parent
Lymph	18.5	####
CD3+	64.4	11.9
CD3+CD8+	20.7	13.4
CD3+CD4+	75.5	48.7
Q1-1	2.8	0.5
CD4+	51.2	9.5
Q3-1	30.8	5.7
Q4-1	15.2	2.8
Q1-2	2.9	0.5
CD8+	16.2	3.0
Q3-2	29.5	5.5
Q4-2	51.5	9.5

BD FACSDiva 8.0.1

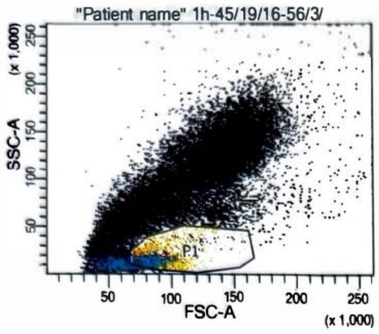

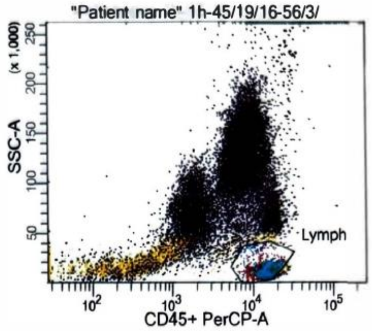

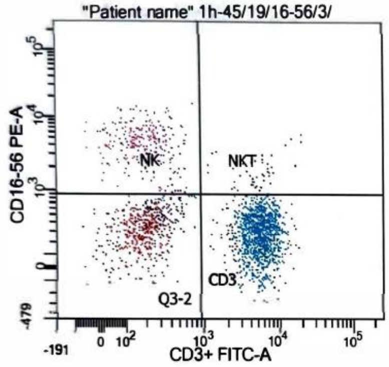

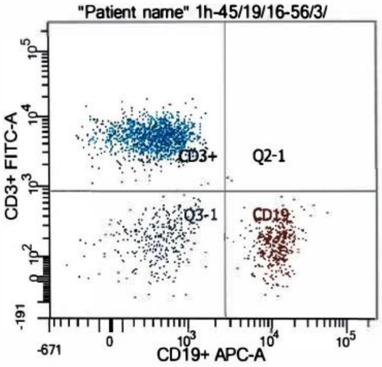

Experiment Name: "Patient name" 1h
Specimen Name: "Patient name" 1h
Tube Name: 45/19/16-56/3/

Population	%Parent
All Events	####
P1	9.5
Lymph	10.3
NK	13.5
NKT	2.7
Q3-2	24.3
CD3	59.5
CD3+	62.2
Q2-1	0.1
Q3-1	17.1
CD19	20.7
P3	58.0
P4	3.4

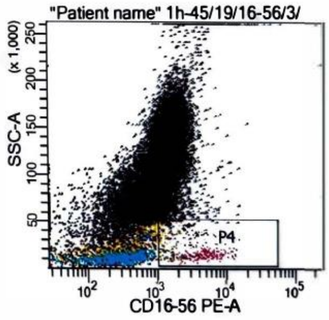

BD FACSDiva 8.0.1

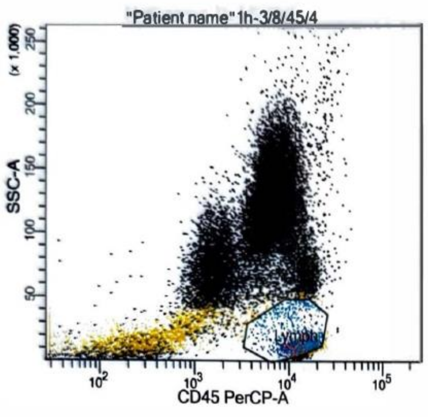

Experiment Name:	"Patient name" 1h
Specimen Name:	"Patient name"1h
Tube Name:	3/8/45/4
Record Date:	May 20, 2022 12:16:29 PM

Population	%Parent	%Grand Parent
Lymph	9.9	####
CD3+	57.1	5.6
CD3+CD8+	18.9	10.8
CD3+CD4+	76.9	43.9
Q1-1	4.0	0.4
CD4+	48.6	4.8
Q3-1	34.0	3.4
Q4-1	13.5	1.3
Q1-2	5.7	0.6
CD8+	17.4	1.7
Q3-2	29.7	2.9
Q4-2	47.3	4.7

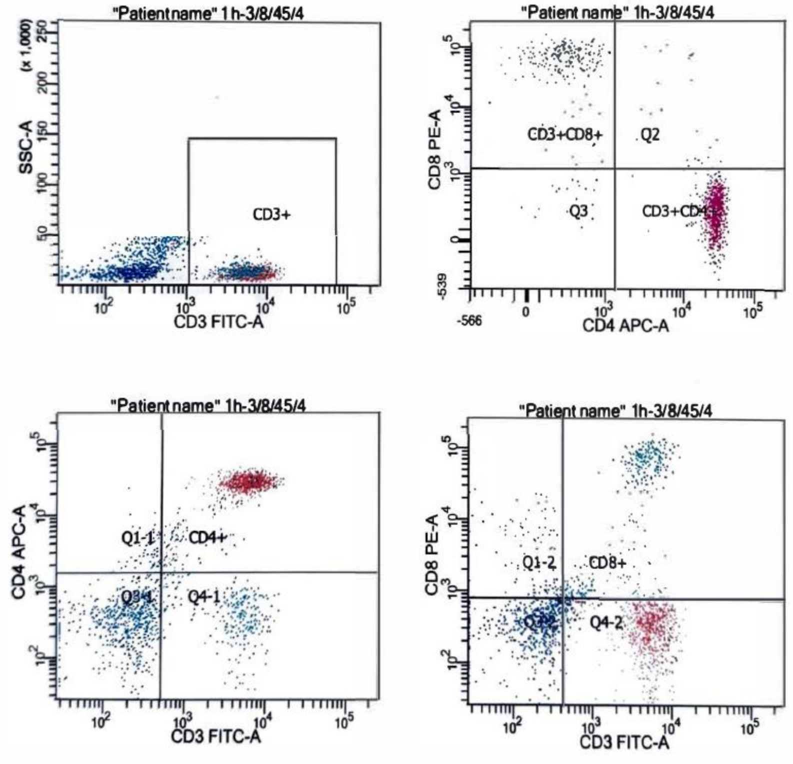

17,4%
1,0[illegible]

3/8/45/4

Printed on: Fri May 20, 2022 12:16:36 PDT

BD FACSDiva 8.0.1

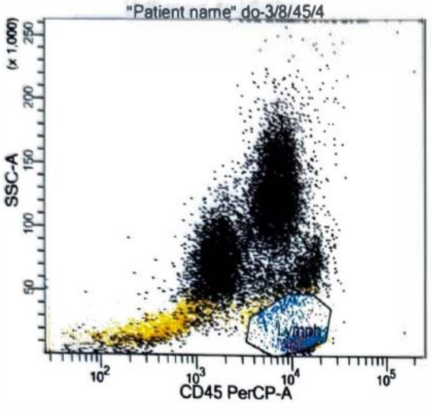

Experiment Name:	"Patient name" do
Specimen Name:	"Patient name" do
Tube Name:	3/8/45/4
Record Date:	May 20, 2022 12:13:18 PM

Population	%Parent	%Grand Parent
Lymph	12.7	####
CD3+	56.9	7.1
CD3+CD8+	18.7	10.5
CD3+CD4+	77.1	43.1
Q1-1	3.7	0.5
CD4+	45.8	5.8
Q3-1	38.4	4.9
Q4-1	12.2	1.5
Q1-2	2.9	0.4
CD8+	13.4	1.7
Q3-2	37.2	4.7
Q4-2	46.5	5.9

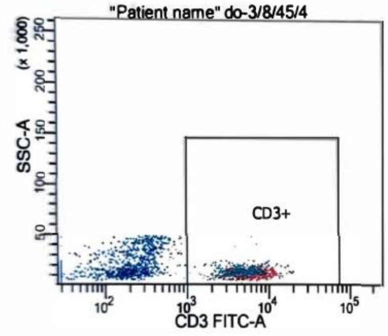

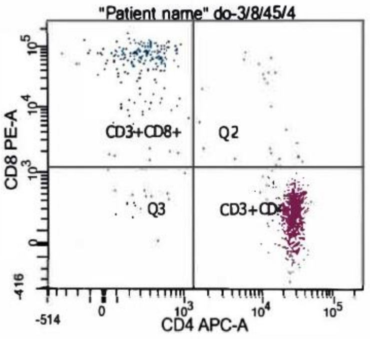

22,3/
1,39

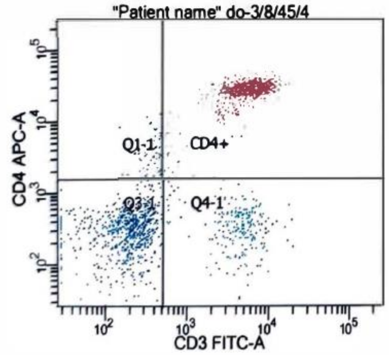

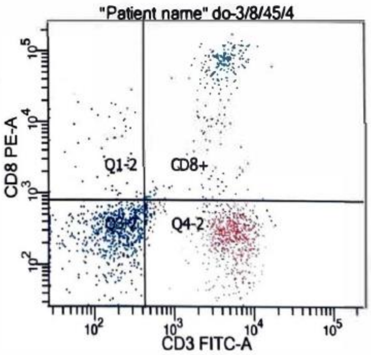

BD FACSDiva 8.0.1

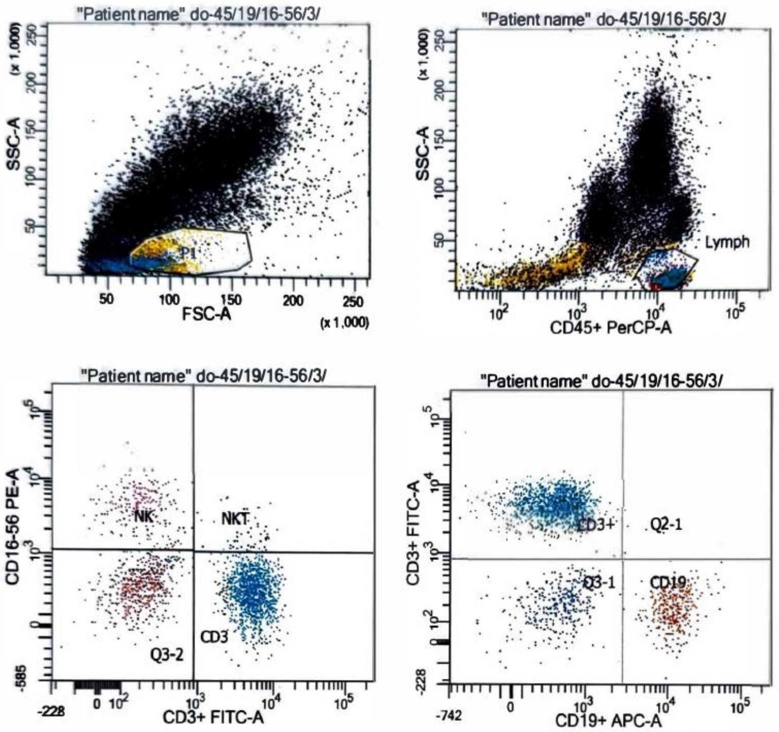

Experiment Name:	"Patient name" do
Specimen Name:	"Patient name" do
Tube Name:	45/19/16-56/3/

Population	%Parent
All Events	####
P1	10.8
Lymph	10.4
NK	12.0
NKT	3.3
Q3-2	25.3
CD3	59.5
CD3+	62.4
Q2-1	0.3
Q3-1	18.3
CD19	19.1
P3	58.0
P4	5.2

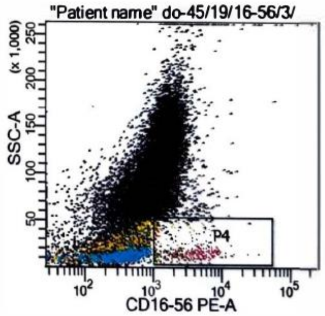

45/19/16-56/3/

Printed on: Fri May 20, 2022 12:14:45 PDT

BD FACSDiva 8.0.1

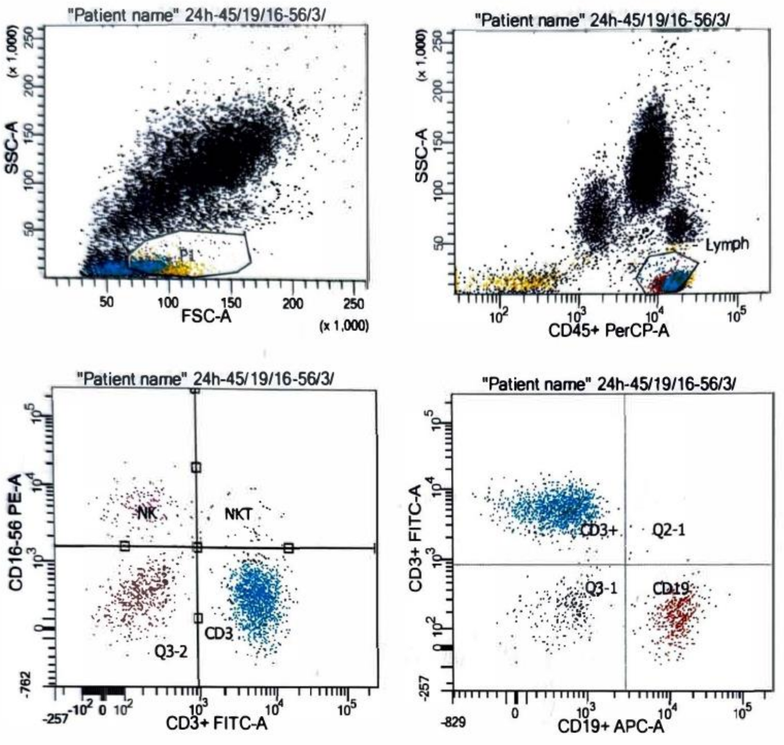

**Experiment Name:**	"Patient name" 24h
**Specimen Name:**	"Patient name" 24h
**Tube Name:**	45/19/16-56/3/

Population	%Parent
All Events	####
P1	13.5
Lymph	17.6
NK	8.5
NKT	2.1
Q3-2	22.7
CD3	66.9
CD3+	68.5
Q2-1	0.5
Q3-1	10.5
CD19	20.5
P3	65.6
P4	4.4

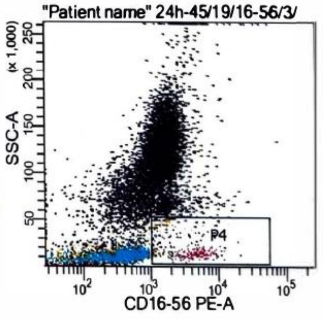

4. Figure S2. Flow Cytometry gating. Patient 2.

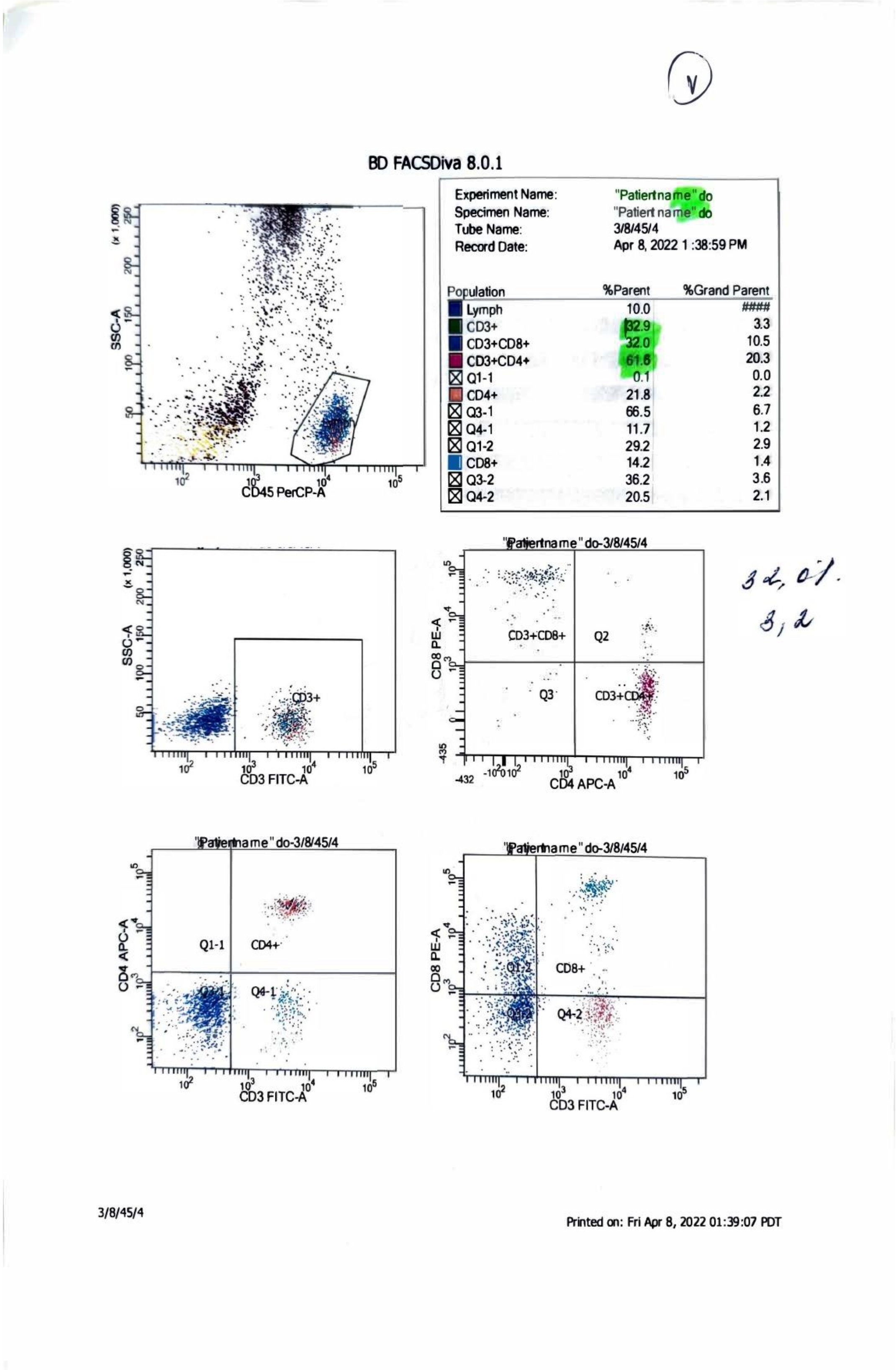

BD FACSDiva 8.0.1

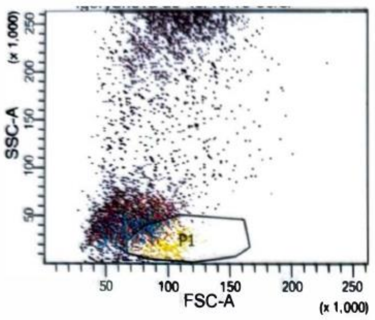

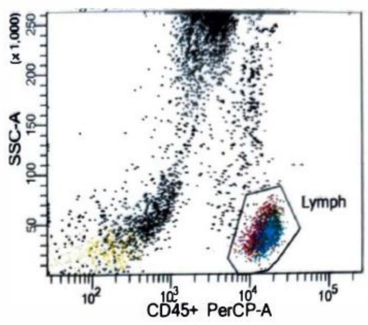

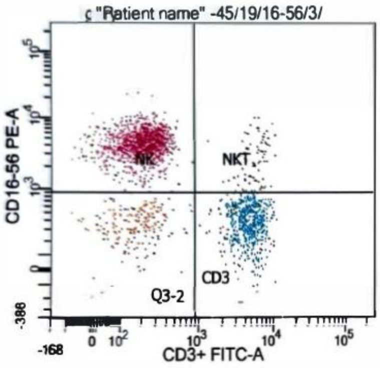

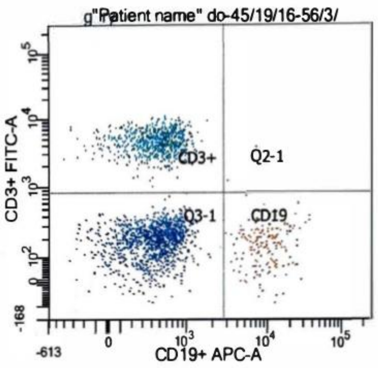

Experiment Name:	"Patient name" do
Specimen Name:	"Patient name" do
Tube Name:	45/19/16-56/3/

Population	%Parent
All Events	####
P1	5.3
Lymph	11.3
NK	56.3
NKT	4.4
Q3-2	9.3
CD3	30.1
CD3+	34.3
Q2-1	0.1
Q3-1	56.7
CD19	8.9
P3	29.6
P4	10.0

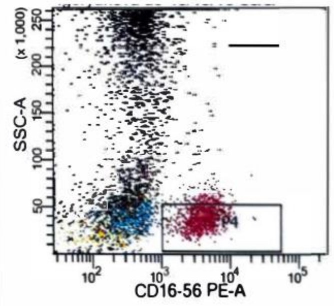

BD FACSDiva 8.0.1

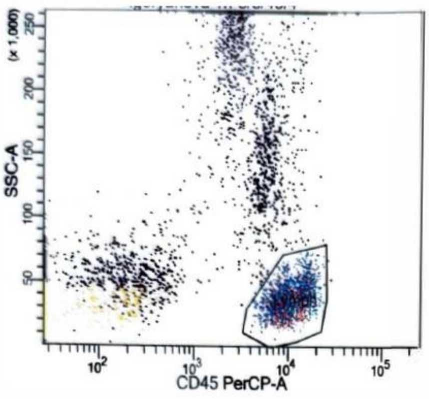

Experiment Name:	"Patient name" 1h
Specimen Name:	"Patient name" 1h
Tube Name:	3/8/45/4
Record Date:	Apr 8, 2022 1:42:10 PM

Population	%Parent	%Grand Parent
Lymph	27.1	####
CD3+	42.3	11.4
CD3+CD8+	26.5	11.2
CD3+CD4+	66.9	28.2
Q1-1	0.0	0.0
CD4+	29.6	8.0
Q3-1	57.5	15.5
Q4-1	13.0	3.5
Q1-2	16.0	4.3
CD8+	13.5	3.7
Q3-2	40.2	10.9
Q4-2	30.3	8.2

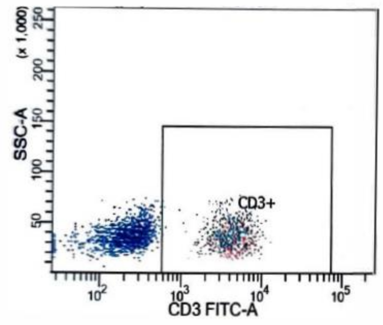

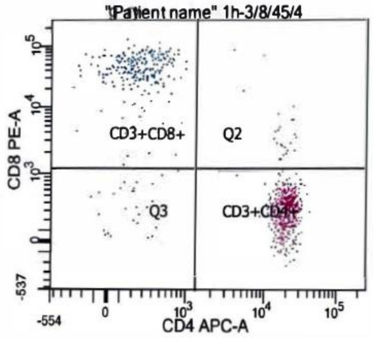

11,0%
1,3

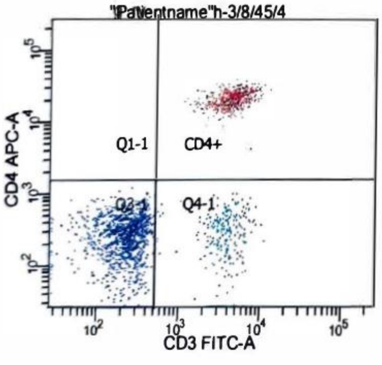

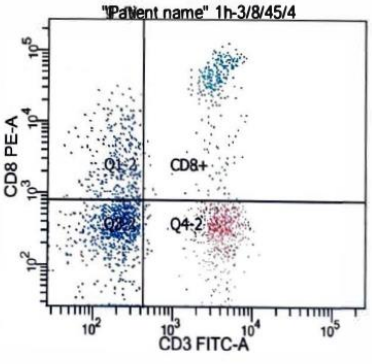

3/8/45/4

Printed on: Fri Apr 8, 2022 01:42:20 PDT

BD FACSDiva 8.0.1

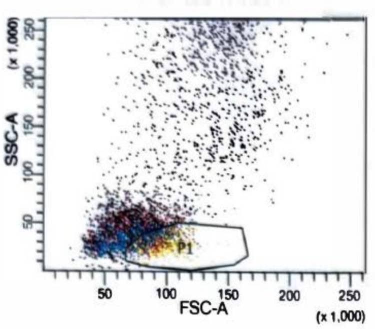

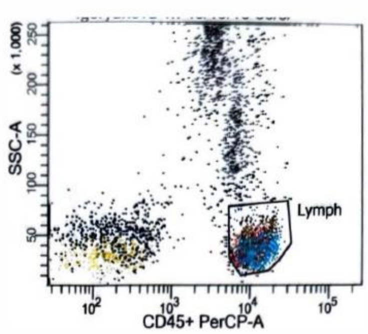

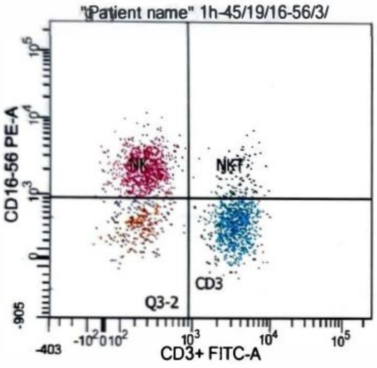

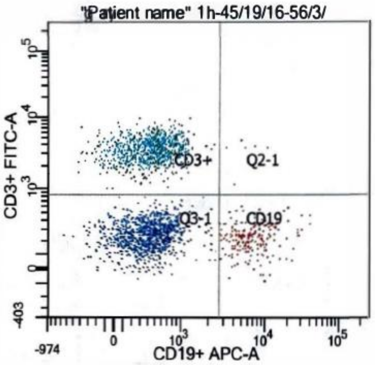

Experiment Name: I"Patient name" 1h
Specimen Name: "Patient name" 1h
Tube Name: 45/19/16-56/3/

Population	%Parent
All Events	####
P1	16.5
Lymph	27.0
NK	44.1
NKT	6.0
Q3-2	13.1
CD3	36.9
CD3+	42.6
Q2-1	0.3
Q3-1	46.7
CD19	10.5
P3	34.9
P4	9.2

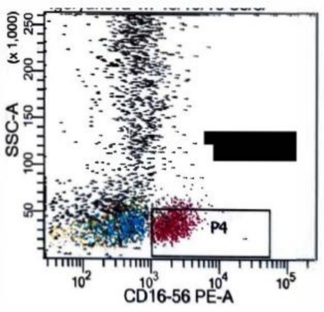

BD FACSDiva 8.0.1

Experiment Name:	"Patient name" 24h
Specimen Name:	"Patient name" 24h
Tube Name:	3/8/45/4
Record Date:	Apr 8, 2022 1:44:17 PM

Population	%Parent	%Grand Parent
Lymph	21.9	####
CD3+	68.2	14.9
CD3+CD8+	24.2	16.5
CD3+CD4+	73.1	49.9
Q1-1	0.3	0.1
CD4+	50.9	11.1
Q3-1	31.4	6.9
Q4-1	17.4	3.8
Q1-2	9.4	2.1
CD8+	18.0	3.9
Q3-2	22.0	4.8
Q4-2	50.6	11.1

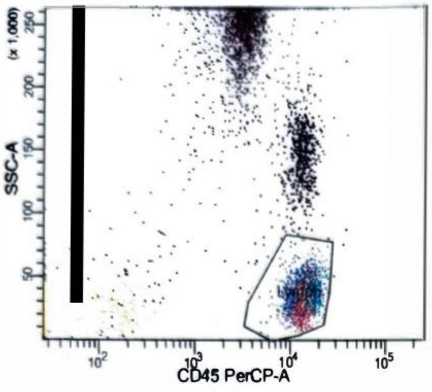

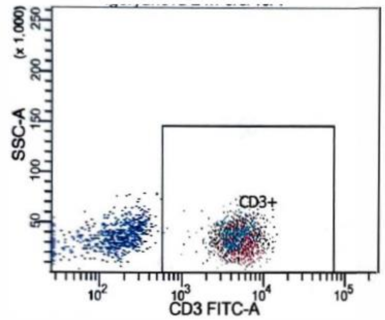

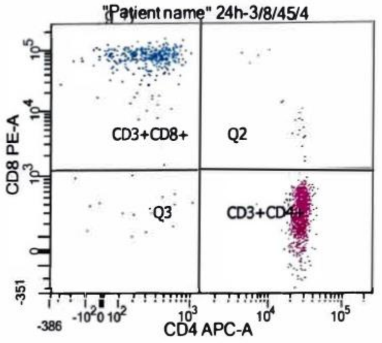

21,5%.
2,8

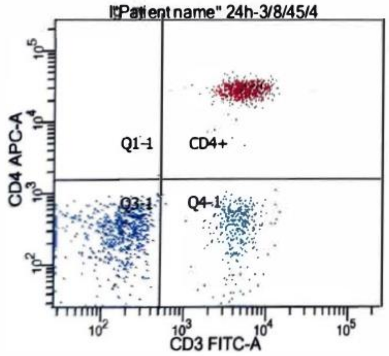

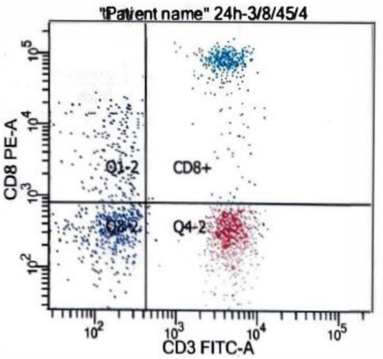

3/8/45/4

Printed on: Fri Apr 8, 2022 01:44:24 PDT

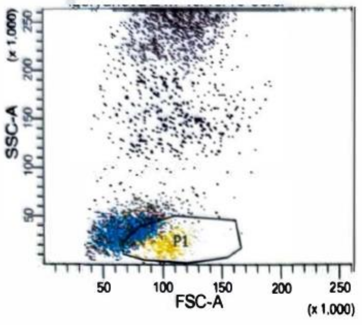

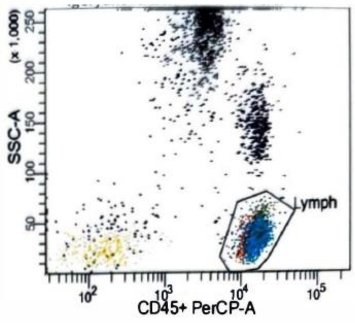

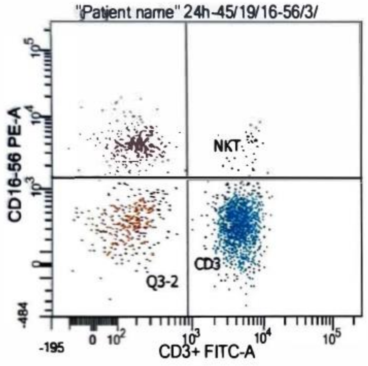

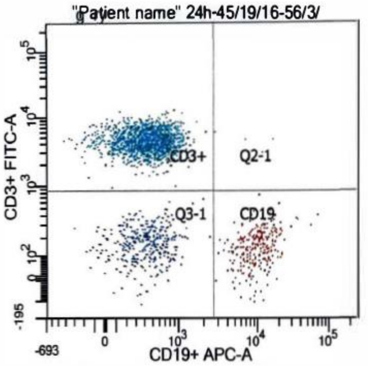

Experiment Name: "Patient name" 24h
Specimen Name: "Patient name" 24h
Tube Name: 45/19/16-56/3/

Population	%Parent
All Events	####
P1	13.6
Lymph	25.0
NK	15.0
NKT	2.1
Q3-2	14.8
CD3	68.2
CD3+	70.1
Q2-1	0.2
Q3-1	16.1
CD19	13.7
P3	64.6
P4	3.4

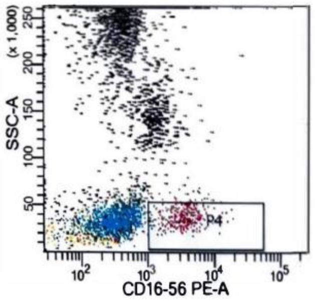

Supplement: Supplementary file 1 [file DataSheet_1.pdf]
